# Assessment of paediatric inpatient care during a multifaceted quality improvement intervention in Kenyan District Hospitals – use of prospectively collected case record data

**DOI:** 10.1186/1472-6963-14-312

**Published:** 2014-07-18

**Authors:** Paul Mwaniki, Philip Ayieko, Jim Todd, Mike English

**Affiliations:** 1 KEMRI/Wellcome Trust Research Programme, Nairobi, Kenya; 2 Department of Population Health, London School of Hygiene and Tropical Medicine, London, UK; 3 Department of Paediatrics, University of Oxford, Oxford, UK

**Keywords:** Quality improvement, Prospective, Retrospective, Paediatrics, Health services research, Kenya

## Abstract

**Background:**

In assessing quality of care in developing countries, retrospectively collected data are usually used given their availability. Retrospective data however suffer from such biases as recall bias and non-response bias. Comparing results obtained using prospectively and retrospectively collected data will help validate the use of the easily available retrospective data in assessing quality of care in past and future studies.

**Methods:**

Prospective and retrospective datasets were obtained from a cluster randomized trial of a multifaceted intervention aimed at improving paediatric inpatient care conducted in eight rural Kenyan district hospitals by improving management of children admitted with pneumonia, malaria and diarrhea and/or dehydration. Four hospitals received a full intervention and four a partial intervention. Data were collected through 3 two weeks surveys conducted at baseline, after 6 and 18 months. Retrospective data was sampled from paediatric medical records of patients discharged in the preceding six months of the survey while prospective data was collected from patients discharged during the two week period of each survey. Risk Differences during post-intervention period of16 quality of care indicators were analyzed separately for prospective and retrospective datasets and later plotted side by side for comparison.

**Results:**

For the prospective data there was strong evidence of an intervention effect for 8 of the indicators and weaker evidence of an effect for one indicator, with magnitude of effect sizes varying from 23% to 60% difference. For the retrospective data, 10 process (these include the 8 indicators found to be statistically significant in prospective data analysis) indicators had statistically significant differences with magnitude of effects varying from 10% to 42%. The bar-graph comparing results from the prospective and retrospective datasets showed similarity in terms of magnitude of effects and statistical significance for all except two indicators.

**Conclusion:**

Multifaceted interventions can help improve adoption of clinical guidelines and hence improve the quality of care. The similar inference reached after analyses based on prospective assessment of case management is a useful finding as it supports the utility of work based on examination of retrospectively assembled case records allowing longer time periods to be studied while constraining costs.

**Trial registration:**

Current Controlled Trials ISRCTN42996612. Trial registration date: 20/11/2008

## Background

Effective organization and provision of care for children admitted to hospital with acute illness in developing countries is advocated through World Health Organization and Kenyan guidance [1-4]. The effectiveness and cost-effectiveness of one approach to their implementation in Kenya is reported [5]. As with other studies evaluation, the study was based on retrospective examination of case records [6,7] that are typically more feasible than examining inpatient care prospectively by observation. The appropriateness of using medical records in epidemiological research depends on the accuracy of transfer of information from patient to clinician, the accuracy and level of detail in recording that information onto medical records by the clinician and how well abstraction of information from medical records is done [8]. The quality of data obtained from medical records can be compromised by unclearly specified research questions, vague specification of variables, poorly designed abstraction tools, poor understanding of the data by the abstractors and incompleteness of the data in medical records [9]. Comparison of self-reported data with data abstracted from health records on initial treatment of prostate cancer showed good agreement between the two datasets [10] while comparison of prospectively collected data with retrospectively collected data in studying risk factors for coronary artery disease showed that medical records are less accurate and less complete compared to prospectively collected data [11]. Eder et al. discusses some of the strategies that can be used in enhancing the reliability of data abstracted for medical records. These include establishment of priority in the source of information if there are multiple sources of information and having standardized terminology [12]. Abstracting data from medical records can be preferable to prospective designs because they are less resource intensive, they can be easily used in exploring possible associations and can be performed at researchers’ convenience. However, such data are subject to numerous sources of bias, usually have a lot of missing data especially where documentation of care is poor and it’s often difficult to establish true causal effect relationships [8]. Here we use prospectively collected case data to examine the effects of an intervention aimed at improving paediatric practices in Kenya and contrast these findings with those previously reported based on retrospectively collected data [6]. Our aims is both to examine the intervention effects using arguably a more robust data set and triangulate the findings with those based on retrospective case record review.

## Methods

### Study sites and participants

Eight rural district hospitals from four of the eight provinces were purposively chosen to represent rural district hospitals in Kenya and have been described in full elsewhere [1]. Neither the Ministry Of Health nor the hospitals had any defined procedures for implementing new clinical guidelines prior to the study. The Kenya Medical Research Institute National Ethics and Scientific review committees approved the study.

### Randomization and masking

Before randomization, meetings were held with the management of the eight shortlisted hospitals in which the study design, mode of data collection, longevity of the study and intervention were discussed. Each hospital held internal discussion after which the study team sought assent regarding the hospital’s participation in the study. After obtaining assent from all eight hospitals, the hospitals were allocated to either the full intervention or partial intervention using restricted randomization. Hospitals coded as H1-H4 received the full intervention while hospitals H5-H8 received a partial intervention (control). Of the 70 possible randomization outcomes to the intervention and control groups, seven gave relatively balanced groups in terms of hospital level covariates, and one of these was randomly chosen using a “blind draw” procedure. It was not possible to mask treatment of participating hospitals, but details on group allocations were not publicly disseminated, geographical distance between hospitals was relatively large and there is typically little formal effort to transfer knowledge and practice between hospitals.

### Study intervention

The intervention was delivered over an 18 month-period (from September 2006 to April 2008) and aimed to improve quality of pediatric admission care through implementation of best practice guidelines and local efforts to tackle organizational constraints. The partial intervention delivered in control hospitals involved a 1.5 day lecture-based introductory seminar explaining the evidence based clinical practice guidelines followed by dissemination of these guidelines and accompanying job aides, and regular hospital performance assessment surveys conducted every 6 months followed by written feedback. Conversely, the intervention hospitals received 5.5 day training on ETAT + [2], a local hospital facilitator responsible for on-site problem solving who received external supervisory support by telephone from the implementation team, and face to face feedback of survey findings at the end of each survey (see [13] for full description). The package delivered in intervention hospitals was in addition to written feedback and clinical guideline and job aide dissemination.

### Data collection

Data relevant to this study were collected at baseline, six months post baseline and at the end of intervention (18 months) in both control and intervention hospitals. Data collection teams received three-weeks training including a pilot survey prior to baseline data collection with further details of procedures supplied elsewhere [13]. At baseline and for each subsequent round up to four data collection teams working concurrently spent two weeks at each hospital collecting retrospective data from a random sample of medical records of children discharged over the preceding six months [6]. During each survey one team member was assigned to collect prospective data on process of care for all children present on the wards at the start of the survey and every child admitted during the two weeks period that followed. Therefore the retrospective dataset covers children admitted during the six months period preceding the survey while the prospective data set covers children admitted during the two weeks period that data collection was taking place. The aim was to enroll 50 cases per survey per hospital based on estimated admission rates for the size of hospital studied. Data were abstracted on standardized forms from medical records and other supporting documents such as nursing charts and laboratory requests with clarification being sought form health workers or children’s caretakers as needed. For quality assurance purposes team leaders assessed data quality for all cases and independently re-evaluated a 10% sample of retrospective and prospective case records during data collection. Ethical approval was granted for confidential abstraction of data from case records without individuals’ consent.

### Performance indicators

The primary outcome was change in quality of pediatric care measured using 13 process of care indicators in intervention versus control hospitals. These process indicators, the same as those used for the retrospective data analysis [6], were derived from evidence based clinical guideline recommendations for management of pneumonia, malaria and diarrhea and/or dehydration. Three additional indicators focusing on key policy recommendations for paediatric care were vitamin A administration on admission, provider initiated HIV testing and identification of missed opportunities for vaccination. Mortality was not a primary outcome.

All the 13 process indicators were dichotomous variables indicating whether the patient assessment, treatment and supportive care were implemented according to guidelines. An overall score for assessment was calculated representing the proportion of relevant assessment tasks completed for each child. This score, constrained between zero and one, was derived from a maximum possible number of assessment indicators for each child, with 5 indicators for all children, and extra indicators for children diagnosed with malaria (4), pneumonia (4) and diarrhea/dehydration (2) (Additional file 1).

### Sample size

The data collection period for each hospital in each survey was restricted to two weeks limiting the number of prospectively observed patient episodes that could be collected to approximately 50 cases per hospital per survey given the workload in the selected hospitals. Since the number of prospective observations was limited, we explored the ability to detect important effect sizes using typical values of power of 80%, 95% precision and with only 4 clusters per arm with intra-class correlation coefficients derived from retrospective data analysis (Additional file 2). For example, assuming 25 malaria cases per site at the 18 months survey and 50% correct management in control hospitals, the differences between intervention and control arms that would seem an unlikely chance finding ranged from greater than 9% to greater than 20% for ICC values of 0.008 and 0.226 respectively (see Additional file 2). Pooling data across the two post intervention surveys would allow identification of smaller apparent differences (but taking no account of multiple comparisons).

### Statistical analysis

Data from the retrospective study were a sub-set of the data from four surveys (including one at 12 months) used by Ayieko et al. [6]. For the prospective study, data entry was conducted independently by two clerks using Microsoft Access databases and verified. For both studies (prospective and retrospective) data analyses were conducted using Stata version 11 [14]. Descriptive sample characteristics were calculated at the hospital level for each survey period, using medians, and inter-quartile range (IQR) for skewed continuous variables and means or proportions with 95% confidence intervals (95% CI) for continuous and binary categorical variables respectively.

The effect of the intervention was assessed in 2 ways for both the retrospective data and the prospective data. The first set of analyses combined data from surveys 2 and 4 (post intervention) for each hospital, and for each process indicator used an unpaired t-test to compare summary measures in each hospital (4 intervention and 4 control), in order to assess the effect of the intervention [15]. Summary measures of each indicator were used to obtain unadjusted risk differences and risk ratios for the effect of the intervention. The adjusted risk ratios and risk differences were calculated using covariate adjusted cluster residuals, whereby a logistic regression model is fitted using only personal (child-level) factors, and residuals computed for each of the 8 hospitals by subtracting observed hospital means from predicted hospital means [15]. These hospital residuals were compared using an unpaired t-test across the 4 intervention and 4 control hospitals.

A second analysis was undertaken using a multi-level logistic regression model for each process indicator, taking into account the clustering at the hospital level. The multi-level model used data from all three surveys, not adjusting for any individual level characteristics, or hospital level characteristics, to obtain crude odds ratios (OR) to assess the impact of the intervention as the interaction between intervention hospitals in survey 2 and 4 pooled together compared to the main effects of the intervention at baseline. This was reported as ratios of odds ratios for dichotomous indicators and differences in differences for assessment score. A bar chart comparing adjusted risk ratios for prospective and retrospective datasets was plotted to provide a visual indication of how well the results from the two datasets agree.

## Results

All eight participating hospitals completed the study providing 1295 admission episodes followed up prospectively. These included 505 episodes from the pre-intervention period and 790 from the post intervention period (413 at 6 months and 377 at 18 months). A total of 6302 retrospective case records were available for analysis from similar surveys. The characteristics of children whose admissions were evaluated prospectively were similar to those in the retrospective dataset, Table 1. Full description of process indicators for both prospective and retrospectively datasets provided in Additional files 3 and 4 respectively show improvement for many of those indicators between baseline and follow up at 6 months and 18 months. No hospital received additional training from external sources or other intervention components during the study period.

**Table 1:** Demographic characteristic of children by survey during prospective and retrospective data collection

	**Control**		**Intervention**	
	**Prospective**	**Retrospective**	**Prospective**	**Retrospective**
Baseline (Survey 1)				
Number of admission episodes included in analysis	253	1005	252	1130
Mean (SD) age (months)	16.2(13.3)	15.8(12.5)	17.0(13.3)	15.8(12.7)
Number (%) Male	101(56.1)	389(55.0)	72(53.3)	241(53.4)
Number (%) of children with structured admission form in medical records	24.0(9.5)	30.0(3.0)	0.0(0.0)	0.0(0.0)
6 month (survey 2)				
Number of admission episodes included in analysis	215	840	198	1022
Mean (SD) age (months)	14.4(12.3)	16.7(13.4)	14.6(12.1)	17.1(13.1)
Number (%) Male	97(50.5)	406(54.1)	88(56.4)	487(56.3)
Number (%) of children with structured admission form in medical records	98.0(46.2)	590.0(70.9)	185.0(96.4)	929.0(91.2)
18 months (survey 4)				
Number of admission episodes included in analysis	178	1147	199	1158
Mean (SD) age (months)	17.4(14.3)	15.7(12.8)	18.2(14.9)	16.2(12.8)
Number (%) Male	93(58.9)	588(53.0)	92(51.1)	639(58.0)
Number (%) of children with structured admission form in medical records	80.0(45.7)	642.0(56.0)	178.0(90.8)	1112.0(96.2)

**Figure 1:**
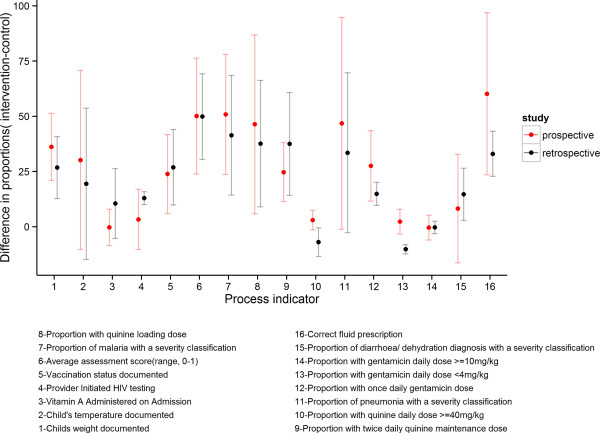
Comparison of prospective and retrospective risk differences.

The adjusted risk difference and risk ratio between intervention and control hospitals for the process indicators obtained from the pooled (6 months plus 18 months) prospective data and for the retrospective data pooled over the same periods are shown in Table 2 (unadjusted results are shown in Table 3). The magnitude of the intervention effect varied across the process indicators. For the prospective data there was strong evidence of an intervention effect for 8 of the indicators and weaker evidence of an effect for one indicator, with magnitude of effect sizes varying from 23% to 60% difference. For the retrospective data, 10 process indicators had statistically significant differences with magnitude of effects varying from 10% to 42%. The mean risk difference between intervention and control hospitals using data collected at six months and 18 months surveys only in both the prospective and retrospective datasets are displayed graphically in Figure 1. This illustrates their similarity in terms of magnitude of effects and statistical significance for all except two indicators, the proportion of patients receiving quinine that received an overdose and the proportion of patients receiving gentamicin that received an under dose. In both of these indicators, the retrospective dataset gave more favorable results. These discrepancies were possibly caused by the small number of patients who had either a gentamicin or quinine prescription on which to calculate these dosage error indicators particularly in the prospective data set.

**Table 2:** Adjusted differences in process of care indicators during the post intervention period

	**Prospective**				**Retrospective**			
**Indicator of quality of care**	**Intervention %**	**Control %**	**Difference [95% CI]**	**Risk ratio [95% CI]**	**Intervention %**	**Control %**	**Difference [95% CI]**	**Risk ratio [95% CI]**
All Children								
Childs weight documented	79.1	45	36.2[21.0-51.4]	1.9[1.4-2.6]	82.8	59.1	26.8[12.8-40.8]	1.5[1.2-1.9]
Child’s temperature documented	63	30.5	30.2[−10.3-70.7]	3.7[0.6-22.4]	64.9	46	19.4[−14.8-53.7]	1.9[0.6-5.8]
Vitamin A Administered on Admission	16.4	17.3	−0.3[−8.5-7.9]	1.2[0.7-2.0]	22.2	11.9	10.5[−5.3-26.3]	2.1[0.5-9.9]
Provider Initiated HIV testing	18.1	15.8	3.3[−10.3-16.9]	1.3[0.5-3.2]	16	3.5	13.0[10.1-15.9]	5.8[2.6-12.7]
Vaccination status documented	41.3	18.6	23.9[6.0-41.7]	3.1[0.9-10.0]	50.8	27.2	26.9[9.9-44.0]	2.6[1.2-5.6]
Average assessment score (range, 0–1)	92.1	58.7	50.1[23.9-76.3]		92.6	67.2	49.9[30.5-69.2]	
Children with diagnosis of malaria								
Proportion of malaria with a severity classification	91.9	43	50.9[23.7-78.0]	2.4[1.3-4.4]	89.1	46.8	41.4[14.3-68.5]	2.0[1.1-3.5]
Proportion with quinine loading dose	88.5	59.3	46.4[5.9-86.8]	2.5[0.9-7.1]	86.9	58.1	37.6[9.0-66.2]	2.0[1.0-3.8]
Proportion with twice daily quinine maintenance dose	94.1	74.9	24.7[11.4-38.1]	1.4[1.1-1.7]	79	42.5	37.5[14.2-60.7]	1.9[1.3-2.8]
Proportion with quinine daily dose > =40 mg/kg	4	1	3.0[−1.4-7.4]		2.2	8	−7.0[−13.4--0.5]	0.2[0.1-0.6]
Children with diagnosis of pneumonia								
Proportion of pneumonia with a severity classification	95.8	63.6	46.8[−1.2-94.8]	1.5[1.1-2.0]	93.3	70.1	33.5[−2.7-69.7]	1.7[0.9-3.1]
Proportion with once daily gentamicin dose	86.8	57.6	27.6[11.6-43.5]	1.5[1.1-2.1]	79.1	68.1	14.9[9.7-20.1]	1.2[1.1-1.4]
Proportion with gentamicin daily dose <4 mg/kg	3.9	3.4	2.3[−3.3-7.9]	1.9[0.8-4.3]	3.9	15.3	−10.2[−12.3--8.1]	0.3[0.2-0.4]
Proportion with gentamicin daily dose > =10 mg/kg	4.7	6.8	−0.4[−6.0-5.2]	1.0[0.5-2.2]	8.6	9.9	−0.3[−3.1-2.5]	1.0[0.7-1.4]
Children with diagnosis of diarrhea/dehydration								
Proportion of diarrhoea/dehydration diagnosis with a severity classification	98.6	85.2	8.2[−16.3-32.8]	1.1[0.8-1.5]	97.9	89.1	14.7[2.9-26.5]	1.2[1.0-1.4]
Correct fluid prescription	67.3	11.5	60.2[23.5-96.9]	2.7[1.4-5.3]	62.2	32.7	33.0[22.8-43.2]	2.1[1.6-2.9]

**Table 3:** Unadjusted differences in process of care indicators during the post intervention period

	**Prospective**				**Retrospective**			
**Indicator of quality of care**	**Intervention %**	**Control %**	**Difference [95% CI]**	**Risk ratio [95% CI]**	**Intervention**	**Control**	**Difference [95% CI]**	**Risk ratio [95% CI]**
All Children								
Childs weight documented	79.1	45	34.1[27.7-40.4]	1.8[1.6-2.0]	82.8	59.1	23.7[21.0-26.4]	1.4[1.3-1.5]
Child’s temperature documented	63	30.5	32.4[25.9-39.0]	2.1[1.7-2.4]	64.9	46	18.9[15.9-21.8]	1.4[1.3-1.5]
Vitamin A Administered on Admission	16.4	17.3	−0.9[−6.1-4.3]	0.9[0.7-1.3]	22.2	11.9	10.3[8.0-12.5]	1.9[1.6-2.1]
Provider Initiated HIV testing	18.1	15.8	2.3[−2.9-7.6]	1.1[0.8-1.6]	16	3.5	12.6[10.8-14.3]	4.6[3.6-6.0]
Vaccination status documented	41.3	18.6	22.7[16.6-28.9]	2.2[1.8-2.8]	50.8	27.2	23.6[20.8-26.5]	1.9[1.7-2.0]
Average assessment score (range, 0–1)	92.1	58.7	33.4[29.9-36.9]		92.6	67.2	25.4[23.9-26.9]	
Children with diagnosis of malaria								
Proportion of malaria with a severity classification	91.9	43	48.9[42.1-55.7]	2.1[1.9-2.5]	89.1	46.8	42.3[39.1-45.5]	1.9[1.8-2.0]
Proportion with quinine loading dose	88.5	59.3	29.2[20.5-37.9]	1.5[1.3-1.7]	86.9	58.1	28.8[24.8-32.8]	1.5[1.4-1.6]
Proportion with twice daily quinine maintenance dose	94.1	74.9	19.2[12.2-26.2]	1.3[1.1-1.4]	79	42.5	36.5[32.0-41.1]	1.9[1.7-2.0]
Proportion with quinine daily dose > =40 mg/kg	4	1	2.9[−0.1-6.0]	3.8[0.8-17.6]	2.2	8	−5.8[−8.0--3.6]	0.3[0.2-0.4]
Children with diagnosis of pneumonia								
Proportion of pneumonia with a severity classification	95.8	63.6	32.2[24.0-40.4]	1.5[1.3-1.7]	93.3	70.1	23.2[19.9-26.6]	1.3[1.3-1.4]
Proportion with once daily gentamicin dose	86.8	57.6	29.2[19.9-38.5]	1.5[1.3-1.7]	79.1	68.1	11.1[6.7-15.4]	1.2[1.1-1.2]
Proportion with gentamicin daily dose <4 mg/kg	3.9	3.4	0.5[−3.8-4.8]	1.1[0.4-3.7]	3.9	15.3	−11.4[−14.3--8.6]	0.3[0.2-0.4]
Proportion with gentamicin daily dose > =10 mg/kg	4.7	6.8	−2.1[−7.3-3.1]	0.7[0.3-1.8]	8.6	9.9	−1.2[−4.1-1.6]	0.9[0.6-1.2]
Children with diagnosis of diarrhea/dehydration								
Proportion of diarrhoea/dehydration diagnosis with a severity classification	98.6	85.2	13.4[3.6-23.3]	1.2[1.0-1.3]	97.9	89.1	8.7[5.9-11.6]	1.1[1.1-1.1]
Correct fluid prescription	67.3	11.5	55.8[37.8-73.8]	5.8[2.0-17.2]	62.2	32.7	29.5[22.9-36.1]	1.9[1.6-2.2]

The results from multilevel models comparing the effect of intervention during the post-intervention period to that at baseline for both prospective and retrospective datasets are presented in Table 4. The prospective data showed statistically significant differences between baseline and post-intervention periods for 8 process indicators. These differences were also observed in the retrospective dataset except for HIV testing that showed favorable results in retrospective data. It was not possible to calculate ratio of odds ratios for one indicator (correct fluid prescription for patients presenting with diarrhoea and/or dehydration) in the prospective dataset due to a small number of observations. Analysis of retrospective data suggested a favorable effect of the intervention for this indicator.

**Table 4:** Effect of training for baseline and post-intervention period in prospective data and retrospective data

	**Prospective**			**Retrospective**		
**Indicator**	**Baseline OR**	**Post intervention OR**	**Ratio of OR**	**Baseline OR**	**Post intervention OR**	**Ratio of OR**
All Children						
Childs weight documented	3.95(1.92-8.15)	4.99(2.51-9.94)	1.26(0.76-2.10)	9.32(3.28-26.46)	4.06(1.45-11.39)	0.44(0.33-0.57)
Child’s temperature documented	0.68(0.07-6.23)	4.09(0.46-36.08)	5.99(3.10-11.57)	0.81(0.09-7.34)	1.75(0.20-15.59)	2.15(1.53-3.03)
Vitamin A Administered on Admission	0.23(0.06-0.93)	1.18(0.40-3.53)	5.08(1.87-13.84)	0.32(0.07-1.55)	2.44(0.53-11.29)	7.61(4.93-11.75)
Provider Initiated HIV testing	13.08(2.63-65.14)	1.21(0.56-2.61)	0.09(0.02-0.42)	2.24(0.97-5.18)	5.65(3.57-8.93)	2.52(1.14-5.57)
Vaccination status documented	0.08(0.02-0.38)	4.56(1.22-17.09)	56.29(22.06-143.58)	0.23(0.06-0.81)	4.38(1.24-15.43)	19.42(14.07-26.81)
Average assessment score (range, 0–1)	Mean = −0.04(−0.17-0.09)	Mean = 0.33(0.20-0.45)	Differences in Means = 0.37(0.32-0.41)	Mean = 0.01(−0.10-0.12)	Mean = 0.27(0.16-0.38)	Differences in Means = 0.26(0.24-0.28)
Children with diagnosis of malaria						
Proportion of malaria with a severity classification	2.92(0.95-8.96)	15.28(5.80-40.27)	5.22(2.05-13.31)	6.10(2.35-15.85)	8.80(4.01-19.28)	1.44(0.77-2.69)
Proportion with quinine loading dose	5.72(0.95-34.50)	7.38(2.22-24.61)	1.29(0.28-5.97)	0.41(0.14-1.19)	6.63(2.47-17.76)	16.24(9.28-28.41)
Proportion with twice daily quinine maintenance dose	4.37(1.07-17.89)	5.68(2.30-14.03)	1.30(0.31-5.48)	0.05(0.01-0.24)	6.45(2.23-18.65)	131.29(37.51-459.47)
Proportion with quinine daily dose > =40 mg/kg	2.68(0.16-44.63)	3.25(0.21-49.51)	1.21(0.09-16.85)	1.45(0.77-2.71)	0.50(0.24-1.02)	0.35(0.17-0.68)
Children with diagnosis of pneumonia						
Proportion of pneumonia with a severity classification	2.00(0.34-11.71)	23.33(3.93-138.58)	11.68(3.40-40.07)	1.72(0.59-4.99)	7.29(2.70-19.66)	4.24(2.40-7.48)
Proportion with once daily gentamicin dose	0.65(0.11-3.93)	6.09(1.05-35.31)	9.34(3.05-28.57)	0.29(0.11-0.76)	1.81(0.95-3.43)	6.24(2.78-14.02)
Proportion with gentamicin daily dose <4 mg/kg	2.76(0.60-12.67)	1.38(0.26-7.48)	0.50(0.10-2.63)	2.20(1.18-4.10)	0.21(0.11-0.41)	0.09(0.05-0.16)
Proportion with gentamicin daily dose > =10 mg/kg	1.54(0.28-8.55)	0.67(0.24-1.84)	0.44(0.06-3.19)	0.44(0.24-0.81)	0.90(0.59-1.38)	2.07(1.07-4.00)
Children with diagnosis of diarrhea/dehydration						
Proportion of diarrhoea/dehydration diagnosis with a severity classification	0.23(0.03-1.59)	12.80(1.42-115.47)	55.99(3.03-1034.44)	0.78(0.47-1.28)	5.85(3.07-11.17)	7.53(3.63-15.62)
Correct fluid prescription	3.9e + 06(0.00- .)	15.81(4.13-60.59)	0.00(0.00- .)	0.18(0.06-0.59)	3.57(1.72-7.42)	19.56(7.04-54.40)

## Discussion

We evaluated a multi-faceted approach to implementation of clinical guidelines aimed at treatment of illnesses that cause most deaths in Kenyan district hospitals. We used data collected by personnel present in the hospital for two week survey periods prior to intervention and 6 and 18 months after intervention. The data showed that prior use of guideline recommended practices for treatment of children with severe illness was poor at baseline. Data further showed marked improvement in adoption of guideline recommended practices in both partial and full intervention groups but improvements were more marked in the full intervention group. It is worth noting that improvements were sustained between 6 to 18 months after initial training despite very high staff turn-over amongst the junior clinicians responsible for much care. Indeed, of 109 clinical staff responsible for attending to the patients sampled as part of the retrospective data analysis in survey 4 in the intervention hospitals, only nine (8.3%) had received any specific formal or ad hoc guideline-related training [6].Retrospective data are more efficiently collected than prospective data, and can cover a wider time period reducing possible effects of seasonal or other temporal variations. However, problems of missing data may be greater when using retrospective case record data, a data quality problem potentially overcome by using prospective collection. As demonstrated in Figure 1, the analysis of prospectively collected data gave similar results to those obtained when using retrospective data. This consistency helps validate, through triangulation, the methods used for retrospectively collecting case record data in assessing the quality of care. The results provide reassurance that assessing practice performance using retrospective sampling is of value.

The underlying hypothesis behind these analyses is that the assessment of quality of care will be similar in retrospective and prospective patients. The analysis recommended by Hayes is robust and simple, which makes it ideal for hospital surveys in countries with limited resources. In this paper we had individual level data allowing the analysis to adjust for confounders that may be associated with the quality of care received by these children. The analysis shows the results from the retrospective and prospective patients gave similar results for the impact of the intervention on quality of care. In the multi-level the assessment of the impact of the intervention is made through an interaction effect between survey time (6 & 18 months vs. baseline at 0 months) and the intervention. While this may have theoretical advantages, it may be more sensitive to small numbers, especially in the estimation of the interaction, and the results were much more varied than with the simpler analysis. The distributional assumptions of multilevel models are difficult to verify when we have few clusters as in our case but these models allow for examination of intervention effect and effect of other covariates simultaneously which is not possible using the method proposed by Hayes.

### Limitations

The main limitation of the study design was the fact that hospitals were not selected at random from a list of all eligible Kenyan hospitals. While primarily for logistical reasons the ability to include only a few hospitals (clusters) undermine notions of generalizability and balance even if random selection and random allocation are used [16]. Masking could clearly not be done and this could have resulted in bias during data collection, arguably particularly during prospective data collection. We however tried to minimize such bias by extensive training in survey methods and use of standard operating procedures. This report also illustrates how we have tried to triangulate findings using different datasets likely subject to different potential biases. Finally as there were only four hospitals in each treatment group, efforts to adjust for baseline characteristics may not have been as successful as we would have liked.

## Conclusion

This study helps strengthen the growing evidence [17,18] that multifaceted interventions can help improve adoption of guidelines and more generally the quality of pediatric care. Carrying out studies of similar intervention packages to improve paediatric care in other low income settings would help strengthen the evidence base most applicable to these settings and provide opportunities for understanding the influence of different contexts on effectiveness [19]. While earlier reports based on retrospectively collected data supported the effectiveness of the intervention examined, the analysis of prospectively collected data described here serves to support those findings while overcoming potential bias inherent to the use of retrospective case record review. The similar inference reached after analyses based on prospective assessment of case management is a useful finding as it supports the utility of work based on examination of retrospectively assembled case records allowing longer time periods to be studied while constraining costs. Further we have been able to develop and apply a suite of analytical methods for assessing the impact of interventions aimed at improving paediatric hospital care that should inform the design and conduct of future studies in this field.

## Abbreviations

ETAT+: Emergency triage assessment and treatment; ICC: Intra-cluster correlation; IQR: Inter-quartile range; CI: Confidence interval.

## Competing interests

There are no competing interests.

## Authors’ contributions

PM performed statistical analysis and drafted the manuscript. PA participated in study design, statistical analysis and drafting of the manuscript. JT was involved in statistical analysis. ME conceived the study, obtained the funding for the project, participated in study design and helped to draft the manuscript. All authors read and approved the final manuscript.

## Pre-publication history

The pre-publication history for this paper can be accessed here:

http://www.biomedcentral.com/1472-6963/14/312/prepub

## Supplementary Material

Additional file 1 Process Indicators with Respective Diagnoses. Click here for file

Additional file 2 **Minimum Detectable Effect Sizes Given Fixed Number of Clusters and Varying Baseline Proportions, ICC, and Cluster Sample Sizes.** The figure shows minimum detectable differences given four clusters per arm and baseline proportions of 0.05, 0.1, 0.3, and 0.5. The intra class correlation coefficients for the quality indicators as estimated from the retrospective data and ranged from 0.08 for proportion on pneumonia cases that had a gentamicin overdose to 0.226 for temperature documentation. This work indicate that for malaria, assuming 25 cases per site per survey and 50% correct management in control hospitals, the difference between intervention and control arms could only be detected if it was greater than 9% and 20% for an ICC of 0.008 and 0.226 respectively. Click here for file

Additional file 3 Process indicators by hospital during each survey, from prospective data. Click here for file

Additional file 4 Process indicators by hospital during each survey, from prospective data. Click here for file
