# Supplementary material for: Assessment of paediatric inpatient care during a multifaceted quality improvement intervention in Kenyan District Hospitals – use of prospectively collected case record data
Source: BMC Health Serv Res. 2014 Jul 18;14:312. doi: 10.1186/1472-6963-14-312 (PMC4110369; doi:10.1186/1472-6963-14-312)
Supplement: Additional file 1 — Process Indicators with Respective Diagnoses. [file 1472-6963-14-312-S1.pdf]

Process Indicators with Respective Diagnoses

Process Indicator	Children with diagnosis of malaria	Children with diagnosis of pneumonia	Children with diagnosis of diarrhea/dehydration
Childs weight documented	√	√	√
Child's temperature documented	√	√	√
Vitamin A Administered on Admission	√	√	√
Provider Initiated HIV testing	√	√	√
Vaccination status documented	√	√	√
Average assessment score(range, 0-1)	√	√	√
Proportion of malaria with a severity classification	√		
Proportion with quinine loading dose	√		
Proportion with twice daily quinine maintenance dose	√		
Proportion with quinine daily dose >=40mg/kg	√		
Proportion of pneumonia with a severity classification		√	
Proportion with once daily gentamicin dose		√	
Proportion with gentamicin daily dose <4mg/kg		√	
Proportion with gentamicin daily dose >=10mg/kg		√	
Proportion of diarrhoea/ dehydration diagnosis with a severity classification			√
Correct fluid prescription			√
