# Supplementary figures and images for: Assessment of paediatric inpatient care during a multifaceted quality improvement intervention in Kenyan District Hospitals – use of prospectively collected case record data
Source: BMC Health Serv Res. 2014 Jul 18;14:312. doi: 10.1186/1472-6963-14-312 (PMC4110369; doi:10.1186/1472-6963-14-312)

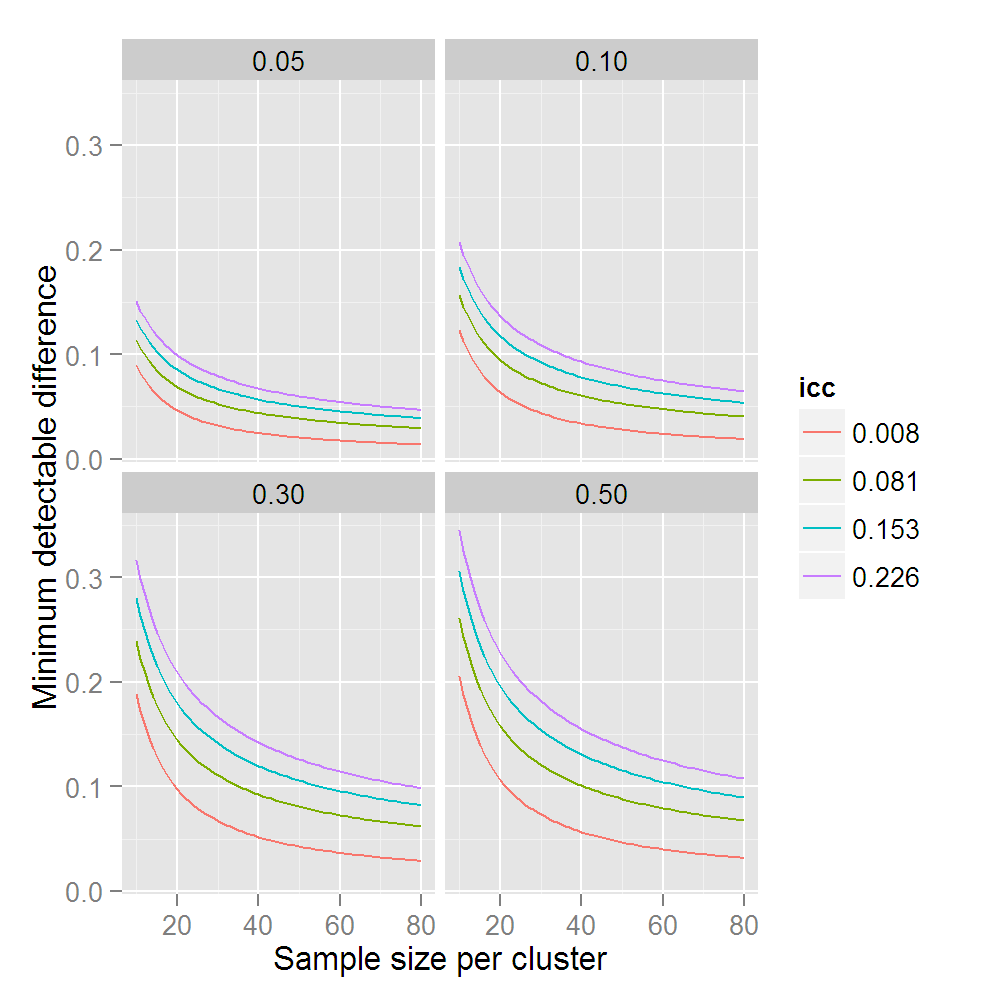

Supplement: Additional file 2 — Minimum Detectable Effect Sizes Given Fixed Number of Clusters and Varying Baseline Proportions, ICC, and Cluster Sample Sizes. The figure shows minimum detectable differences given four clusters per arm and baseline proportions of 0.05, 0.1, 0.3, and 0.5. The intra class correlation coefficients for the quality indicators as estimated from the retrospective data and ranged from 0.08 for proportion on pneumonia cases that had a gentamicin overdose to 0.226 for temperature documentation. This work indicate that for malaria, assuming 25 cases per site per survey and 50% correct management in control hospitals, the difference between intervention and control arms could only be detected if it was greater than 9% and 20% for an ICC of 0.008 and 0.226 respectively. [file 1472-6963-14-312-S2.tiff]
