# Supplementary material for: Assessment of paediatric inpatient care during a multifaceted quality improvement intervention in Kenyan District Hospitals – use of prospectively collected case record data
Source: BMC Health Serv Res. 2014 Jul 18;14:312. doi: 10.1186/1472-6963-14-312 (PMC4110369; doi:10.1186/1472-6963-14-312)
Supplement: Additional file 3 — Process indicators by hospital during each survey, from prospective data. [file 1472-6963-14-312-S3.docx]

Process indicators by hospital during each survey, from prospective data

| **Process indicators** | **H1** | **H2** | **H3** | **H4** | **H5** | **H6** | **H7** | **H8** |
| --- | --- | --- | --- | --- | --- | --- | --- | --- |
|  | n/N(%[95%CI]) | n/N(%[95%CI]) | n/N(%[95%CI]) | n/N(%[95%CI]) | n/N(%[95%CI]) | n/N(%[95%CI]) | n/N(%[95%CI]) | n/N(%[95%CI]) |
| Childs weight documented |  |  |  |  |  |  |  |  |
| Survey1 | 28/55(51[37,65]) | 26/51(51[37,65]) | 66/96(69[58,78]) | 28/50(56[41,70]) | 37/89(42[31,53]) | 8/37(22[10,38]) | 17/65(26[16,39]) | 3/62( 5[ 1,13]) |
| Survey2 | 30/51(59[44,72]) | 43/50(86[73,94]) | 45/47(96[85,99]) | 34/50(68[53,80]) | 13/60(22[12,34]) | 18/51(35[22,50]) | 36/55(65[51,78]) | 23/49(47[33,62]) |
| Survey4 | 41/48(85[72,94]) | 49/55(89[78,96]) | 46/50(92[81,98]) | 26/46(57[41,71]) | 21/51(41[28,56]) | 17/28(61[41,78]) | 35/50(70[55,82]) | 14/49(29[17,43]) |
| Child's temperature documented |  |  |  |  |  |  |  |  |
| Survey1 | 1/55( 2[ 0,10]) | 15/51(29[17,44]) | 1/96( 1[ 0, 6]) | 28/50(56[41,70]) | 6/89( 7[ 3,14]) | 34/37(92[78,98]) | 9/65(14[ 7,25]) | 4/62( 6[ 2,16]) |
| Survey2 | 14/51(27[16,42]) | 34/50(68[53,80]) | 36/47(77[62,88]) | 39/50(78[64,88]) | 1/60( 2[ 0, 9]) | 48/51(94[84,99]) | 16/55(29[18,43]) | 17/49(35[22,50]) |
| Survey4 | 20/48(42[28,57]) | 51/55(93[82,98]) | 32/50(64[49,77]) | 24/46(52[37,67]) | 1/51( 2[ 0,10]) | 24/28(86[67,96]) | 4/50( 8[ 2,19]) | 9/49(18[ 9,32]) |
| Vitamin A Administered on Admission |  |  |  |  |  |  |  |  |
| Survey1 | 2/55( 4[ 0,13]) | 1/51( 2[ 0,10]) | 1/96( 1[ 0, 6]) | 2/50( 4[ 0,14]) | 2/89( 2[ 0, 8]) | 3/37( 8[ 2,22]) | 22/65(34[23,47]) | 0/62( 0[ 0, 6]) |
| Survey2 | 4/51( 8[ 2,19]) | 1/50( 2[ 0,11]) | 3/47( 6[ 1,18]) | 1/50( 2[ 0,11]) | 1/60( 2[ 0, 9]) | 0/51( 0[ 0, 7]) | 26/55(47[34,61]) | 4/49( 8[ 2,20]) |
| Survey4 | 9/48(19[ 9,33]) | 16/55(29[18,43]) | 24/50(48[34,63]) | 7/46(15[ 6,29]) | 7/51(14[ 6,26]) | 10/28(36[19,56]) | 15/50(30[18,45]) | 5/49(10[ 3,22]) |
| Provider Initiated HIV testing |  |  |  |  |  |  |  |  |
| Survey1 | 6/55(11[ 4,22]) | 2/51( 4[ 0,13]) | 14/96(15[ 8,23]) | 2/49( 4[ 0,14]) | 1/89( 1[ 0, 6]) | 0/34( 0[ 0,10]) | 1/65( 2[ 0, 8]) | 0/62( 0[ 0, 6]) |
| Survey2 | 4/49( 8[ 2,20]) | 4/50( 8[ 2,19]) | 9/47(19[ 9,33]) | 4/50( 8[ 2,19]) | 8/60(13[ 6,25]) | 10/48(21[10,35]) | 12/55(22[12,35]) | 3/49( 6[ 1,17]) |
| Survey4 | 18/46(39[25,55]) | 21/55(38[25,52]) | 8/50(16[ 7,29]) | 3/45( 7[ 1,18]) | 4/51( 8[ 2,19]) | 3/26(12[ 2,30]) | 17/49(35[22,50]) | 4/49( 8[ 2,20]) |
| Vaccination status documented |  |  |  |  |  |  |  |  |
| Survey1 | 2/55( 4[ 0,13]) | 3/51( 6[ 1,16]) | 0/96( 0[ 0, 4]) | 1/50( 2[ 0,11]) | 1/89( 1[ 0, 6]) | 4/37(11[ 3,25]) | 30/65(46[34,59]) | 32/62(52[39,65]) |
| Survey2 | 8/51(16[ 7,29]) | 25/50(50[36,64]) | 6/47(13[ 5,26]) | 14/50(28[16,42]) | 1/60( 2[ 0, 9]) | 4/51( 8[ 2,19]) | 18/55(33[21,47]) | 19/49(39[25,54]) |
| Survey4 | 23/48(48[33,63]) | 33/55(60[46,73]) | 38/50(76[62,87]) | 17/46(37[23,52]) | 3/51( 6[ 1,16]) | 5/28(18[ 6,37]) | 14/50(28[16,42]) | 9/49(18[ 9,32]) |
| Average assessment score(range, 0-1) |  |  |  |  |  |  |  |  |
| Survey1 | 31[28,35] | 30[26,35] | 33[29,36] | 33[30,36] | 25[22,28] | 51[42,60] | 41[38,45] | 30[26,34] |
| Survey2 | 90[85,95] | 94[89,98] | 92[89,96] | 90[85,95] | 29[26,33] | 57[50,64] | 68[58,78] | 86[80,92] |
| Survey4 | 84[77,91] | 97[96,99] | 99[97,101] | 89[85,94] | 52[44,60] | 56[44,67] | 42[38,47] | 87[82,92] |
| Proportion of malaria with a severity classification |  |  |  |  |  |  |  |  |
| Survey1 | 14/49(29[17,43]) | 9/26(35[17,56]) | 3/44( 7[ 1,19]) | 1/34( 3[ 0,15]) | 2/71( 3[ 0,10]) | 3/22(14[ 3,35]) | 5/50(10[ 3,22]) | 1/28( 4[ 0,18]) |
| Survey2 | 38/39(97[87,100]) | 32/34(94[80,99]) | 22/27(81[62,94]) | 35/39(90[76,97]) | 8/51(16[ 7,29]) | 11/31(35[19,55]) | 27/37(73[56,86]) | 19/26(73[52,88]) |
| Survey4 | 37/39(95[83,99]) | 32/36(89[74,97]) | 20/22(91[71,99]) | 32/34(94[80,99]) | 17/41(41[26,58]) | 2/15(13[ 2,40]) | 13/39(33[19,50]) | 16/23(70[47,87]) |
| Proportion with quinine loading dose |  |  |  |  |  |  |  |  |
| Survey1 | 15/24(63[41,81]) | 0/1( 0[ 0,98]) | 0/3( 0[ 0,71]) | 9/10(90[55,100]) | 1/3(33[ 1,91]) | 3/4(75[19,99]) | 0/5( 0[ 0,52]) | 0/1( 0[ 0,98]) |
| Survey2 | 34/38(89[75,97]) | 12/15(80[52,96]) | 20/23(87[66,97]) | 23/28(82[63,94]) | 28/31(90[74,98]) | 3/13(23[ 5,54]) | 19/28(68[48,84]) | 2/17(12[ 1,36]) |
| Survey4 | 29/33(88[72,97]) | 28/29(97[82,100]) | 14/16(88[62,98]) | 32/35(91[77,98]) | 22/34(65[46,80]) | 2/4(50[ 7,93]) | 18/22(82[60,95]) | 2/13(15[ 2,45]) |
| Proportion with twice daily quinine maintenance dose |  |  |  |  |  |  |  |  |
| Survey1 | 37/39(95[83,99]) | 3/3(100[29,100]) | 31/31(100[89,100]) | 31/32(97[84,100]) | 67/69(97[90,100]) | 11/17(65[38,86]) | 38/44(86[73,95]) | 19/21(90[70,99]) |
| Survey2 | 33/37(89[75,97]) | 11/11(100[72,100]) | 20/21(95[76,100]) | 30/30(100[88,100]) | 31/40(77[62,89]) | 16/18(89[65,99]) | 15/28(54[34,72]) | 11/17(65[38,86]) |
| Survey4 | 26/28(93[76,99]) | 27/29(93[77,99]) | 13/13(100[75,100]) | 30/33(91[76,98]) | 33/37(89[75,97]) | 5/5(100[48,100]) | 21/35(60[42,76]) | 11/11(100[72,100]) |
| Proportion with quinine daily dose >=40mg/kg |  |  |  |  |  |  |  |  |
| Survey1 | 0/39( 0[ 0, 9]) | 0/3( 0[ 0,71]) | 0/31( 0[ 0,11]) | 2/32( 6[ 1,21]) | 0/69( 0[ 0, 5]) | 1/17( 6[ 0,29]) | 1/44( 2[ 0,12]) | 0/21( 0[ 0,16]) |
| Survey2 | 0/37( 0[ 0, 9]) | 0/11( 0[ 0,28]) | 3/21(14[ 3,36]) | 2/30( 7[ 1,22]) | 0/40( 0[ 0, 9]) | 1/18( 6[ 0,27]) | 0/28( 0[ 0,12]) | 0/17( 0[ 0,20]) |
| Survey4 | 2/28( 7[ 1,24]) | 0/29( 0[ 0,12]) | 0/13( 0[ 0,25]) | 0/33( 0[ 0,11]) | 0/37( 0[ 0, 9]) | 0/5( 0[ 0,52]) | 0/35( 0[ 0,10]) | 0/11( 0[ 0,28]) |
| Proportion of pneumonia with a severity classification |  |  |  |  |  |  |  |  |
| Survey1 | 1/14( 7[ 0,34]) | 19/22(86[65,97]) | 3/39( 8[ 2,21]) | 1/15( 7[ 0,32]) | 4/15(27[ 8,55]) | 9/17(53[28,77]) | 4/26(15[ 4,35]) | 0/38( 0[ 0, 9]) |
| Survey2 | 17/18(94[73,100]) | 31/31(100[89,100]) | 20/21(95[76,100]) | 32/34(94[80,99]) | 0/11( 0[ 0,28]) | 16/32(50[32,68]) | 12/15(80[52,96]) | 27/29(93[77,99]) |
| Survey4 | 8/9(89[52,100]) | 36/38(95[82,99]) | 18/19(95[74,100]) | 19/19(100[82,100]) | 0/4( 0[ 0,60]) | 7/17(41[18,67]) | 9/18(50[26,74]) | 25/25(100[86,100]) |
| Proportion with once daily gentamicin dose |  |  |  |  |  |  |  |  |
| Survey1 | 0/21( 0[ 0,16]) | 0/36( 0[ 0,10]) | 42/65(65[52,76]) | 4/25(16[ 5,36]) | 0/15( 0[ 0,22]) | 5/24(21[ 7,42]) | 25/30(83[65,94]) | 0/43( 0[ 0, 8]) |
| Survey2 | 7/9(78[40,97]) | 21/26(81[61,93]) | 9/10(90[55,100]) | 26/30(87[69,96]) | 15/28(54[34,72]) | 9/33(27[13,46]) | 7/10(70[35,93]) | 27/37(73[56,86]) |
| Survey4 | 10/11(91[59,100]) | 21/22(95[77,100]) | 4/5(80[28,99]) | 14/16(88[62,98]) | 12/14(86[57,98]) | 8/17(47[23,72]) | 9/14(64[35,87]) | 15/24(63[41,81]) |
| Proportion with gentamicin daily dose <4mg/kg |  |  |  |  |  |  |  |  |
| Survey1 | 5/21(24[ 8,47]) | 3/36( 8[ 2,22]) | 3/65( 5[ 1,13]) | 4/25(16[ 5,36]) | 5/15(33[12,62]) | 0/24( 0[ 0,14]) | 0/30( 0[ 0,12]) | 0/43( 0[ 0, 8]) |
| Survey2 | 1/9(11[ 0,48]) | 0/26( 0[ 0,13]) | 1/10(10[ 0,45]) | 2/30( 7[ 1,22]) | 1/28( 4[ 0,18]) | 2/33( 6[ 1,20]) | 0/10( 0[ 0,31]) | 2/37( 5[ 1,18]) |
| Survey4 | 1/11( 9[ 0,41]) | 0/22( 0[ 0,15]) | 0/5( 0[ 0,52]) | 0/16( 0[ 0,21]) | 1/14( 7[ 0,34]) | 0/17( 0[ 0,20]) | 0/14( 0[ 0,23]) | 0/24( 0[ 0,14]) |
| Proportion with gentamicin daily dose >=10mg/kg |  |  |  |  |  |  |  |  |
| Survey1 | 0/21( 0[ 0,16]) | 0/36( 0[ 0,10]) | 2/65( 3[ 0,11]) | 2/25( 8[ 1,26]) | 0/15( 0[ 0,22]) | 0/24( 0[ 0,14]) | 2/30( 7[ 1,22]) | 0/43( 0[ 0, 8]) |
| Survey2 | 0/9( 0[ 0,34]) | 1/26( 4[ 0,20]) | 1/10(10[ 0,45]) | 0/30( 0[ 0,12]) | 2/28( 7[ 1,24]) | 1/33( 3[ 0,16]) | 1/10(10[ 0,45]) | 3/37( 8[ 2,22]) |
| Survey4 | 2/11(18[ 2,52]) | 1/22( 5[ 0,23]) | 1/5(20[ 1,72]) | 0/16( 0[ 0,21]) | 1/14( 7[ 0,34]) | 3/17(18[ 4,43]) | 1/14( 7[ 0,34]) | 0/24( 0[ 0,14]) |
| Proportion of diarrhoea/ dehydration diagnosis with a severity classification |  |  |  |  |  |  |  |  |
| Survey1 | 3/3(100[29,100]) | 0/4( 0[ 0,60]) | 12/13(92[64,100]) | 2/2(100[16,100]) | 3/4(75[19,99]) | 11/11(100[72,100]) | 2/3(67[ 9,99]) | 11/11(100[72,100]) |
| Survey2 | 12/12(100[74,100]) | 11/11(100[72,100]) | 9/9(100[66,100]) | 3/3(100[29,100]) | 5/5(100[48,100]) | 8/9(89[52,100]) | 3/4(75[19,99]) | 3/3(100[29,100]) |
| Survey4 | 4/5(80[28,99]) | 9/9(100[66,100]) | 16/16(100[79,100]) | 8/8(100[63,100]) | 5/5(100[48,100]) | 5/7(71[29,96]) | 6/6(100[54,100]) | 11/15(73[45,92]) |
| Correct fluid prescription |  |  |  |  |  |  |  |  |
| Survey1 | 0/1( 0[ 0,98]) | 1/1(100[ 3,100]) | 0/16( 0[ 0,21]) | 1/2(50[ 1,99]) | 0/0( .[ ., .]) | 0/0( .[ ., .]) | 0/3( 0[ 0,71]) | 0/3( 0[ 0,71]) |
| Survey2 | 6/11(55[23,83]) | 5/7(71[29,96]) | 3/4(75[19,99]) | 1/2(50[ 1,99]) | 1/3(33[ 1,91]) | 0/3( 0[ 0,71]) | 2/9(22[ 3,60]) | 0/1( 0[ 0,98]) |
| Survey4 | 5/7(71[29,96]) | 5/6(83[36,100]) | 4/7(57[18,90]) | 4/5(80[28,99]) | 0/2( 0[ 0,84]) | 0/2( 0[ 0,84]) | 0/4( 0[ 0,60]) | 0/2( 0[ 0,84]) |
