# Supplementary material for: Assessment of paediatric inpatient care during a multifaceted quality improvement intervention in Kenyan District Hospitals – use of prospectively collected case record data
Source: BMC Health Serv Res. 2014 Jul 18;14:312. doi: 10.1186/1472-6963-14-312 (PMC4110369; doi:10.1186/1472-6963-14-312)
Supplement: Additional file 4 — Process indicators by hospital during each survey, from prospective data. [file 1472-6963-14-312-S4.docx]

Process indicators by hospital during each survey, from retrospective data

| Process indicators | H1 | H2 | H3 | H4 | H5 | H6 | H7 | H8 |
| --- | --- | --- | --- | --- | --- | --- | --- | --- |
|  | n/N(%[95%CI]) | n/N(%[95%CI]) | n/N(%[95%CI]) | n/N(%[95%CI]) | n/N(%[95%CI]) | n/N(%[95%CI]) | n/N(%[95%CI]) | n/N(%[95%CI]) |
| Childs weight documented |  |  |  |  |  |  |  |  |
| Survey1 | 113/246(46[40,52]) | 103/330(31[26,37]) | 247/261(95[91,97]) | 192/293(66[60,71]) | 59/121(49[40,58]) | 9/285( 3[ 1, 6]) | 86/276(31[26,37]) | 3/323( 1[ 0, 3]) |
| Survey2 | 181/241(75[69,80]) | 260/331(79[74,83]) | 234/247(95[91,97]) | 154/203(76[69,82]) | 70/108(65[55,74]) | 77/208(37[30,44]) | 205/279(73[68,79]) | 112/245(46[39,52]) |
| Survey3 | 176/231(76[70,82]) | 318/335(95[92,97]) | 263/272(97[94,98]) | 149/236(63[57,69]) | 34/138(25[18,33]) | 101/217(47[40,53]) | 193/232(83[78,88]) | 119/242(49[43,56]) |
| Survey4 | 201/230(87[82,91]) | 304/312(97[95,99]) | 286/309(93[89,95]) | 186/307(61[55,66]) | 123/271(45[39,52]) | 163/198(82[76,87]) | 269/341(79[74,83]) | 156/337(46[41,52]) |
| Child's temperature documented |  |  |  |  |  |  |  |  |
| Survey1 | 7/246( 3[ 1, 6]) | 37/330(11[ 8,15]) | 10/261( 4[ 2, 7]) | 87/293(30[25,35]) | 2/121( 2[ 0, 6]) | 251/285(88[84,92]) | 23/276( 8[ 5,12]) | 7/323( 2[ 1, 4]) |
| Survey2 | 48/241(20[15,26]) | 194/331(59[53,64]) | 184/247(74[69,80]) | 142/203(70[63,76]) | 27/108(25[17,34]) | 185/208(89[84,93]) | 133/279(48[42,54]) | 72/245(29[24,36]) |
| Survey3 | 50/231(22[17,28]) | 275/335(82[78,86]) | 166/272(61[55,67]) | 172/236(73[67,78]) | 11/138( 8[ 4,14]) | 195/217(90[85,94]) | 94/232(41[34,47]) | 109/242(45[39,52]) |
| Survey4 | 125/230(54[48,61]) | 280/312(90[86,93]) | 233/309(75[70,80]) | 209/307(68[63,73]) | 3/271( 1[ 0, 3]) | 187/198(94[90,97]) | 180/341(53[47,58]) | 128/337(38[33,43]) |
| Average assessment score† (range, 0-1) |  |  |  |  |  |  |  |  |
| Survey1 | 30[29,32] | 30[29,31] | 34[33,36] | 31[30,32] | 26[23,28] | 32[31,34] | 44[42,45] | 26[24,27] |
| Survey2 | 85[82,88] | 93[91,95] | 97[96,99] | 88[84,92] | 74[68,81] | 54[50,57] | 71[67,74] | 83[81,85] |
| Survey3 | 84[81,87] | 98[97,98] | 93[91,95] | 92[90,94] | 33[29,37] | 50[46,54] | 77[74,81] | 87[85,88] |
| Survey4 | 88[85,91] | 97[96,98] | 97[96,98] | 92[91,93] | 38[35,40] | 72[67,76] | 65[62,68] | 84[82,85] |
| Proportion of malaria with a severity classification |  |  |  |  |  |  |  |  |
| Survey1 | 46/214(22[16,28]) | 18/219( 8[ 5,13]) | 9/211( 4[ 2, 8]) | 14/220( 6[ 4,10]) | 5/103( 5[ 2,11]) | 4/185( 2[ 1, 5]) | 2/249( 1[ 0, 3]) | 3/142( 2[ 0, 6]) |
| Survey2 | 161/185(87[81,92]) | 161/208(77[71,83]) | 144/151(95[91,98]) | 132/160(82[76,88]) | 57/82(70[58,79]) | 84/150(56[48,64]) | 143/212(67[61,74]) | 66/96(69[58,78]) |
| Survey3 | 153/175(87[82,92]) | 221/239(92[88,95]) | 179/192(93[89,96]) | 163/176(93[88,96]) | 13/61(21[12,34]) | 40/132(30[23,39]) | 115/186(62[54,69]) | 79/114(69[60,78]) |
| Survey4 | 176/184(96[92,98]) | 194/200(97[94,99]) | 163/196(83[77,88]) | 229/243(94[91,97]) | 22/212(10[ 7,15]) | 56/105(53[43,63]) | 60/273(22[17,27]) | 100/127(79[71,85]) |
| Proportion with quinine loading dose |  |  |  |  |  |  |  |  |
| Survey1 | 6/162( 4[ 1, 8]) | 5/84( 6[ 2,13]) | 3/169( 2[ 0, 5]) | 11/205( 5[ 3, 9]) | 7/104( 7[ 3,13]) | 55/105(52[42,62]) | 0/236( 0[ 0, 2]) | 0/125( 0[ 0, 3]) |
| Survey2 | 149/172(87[81,91]) | 82/92(89[81,95]) | 123/131(94[88,97]) | 77/127(61[52,69]) | 58/82(71[60,80]) | 12/84(14[ 8,24]) | 116/193(60[53,67]) | 9/60(15[ 7,27]) |
| Survey3 | 129/148(87[81,92]) | 171/181(94[90,97]) | 57/60(95[86,99]) | 126/156(81[74,87]) | 30/94(32[23,42]) | 20/93(22[14,31]) | 102/192(53[46,60]) | 11/78(14[ 7,24]) |
| Survey4 | 149/168(89[83,93]) | 160/163(98[95,100]) | 86/88(98[92,100]) | 181/218(83[77,88]) | 112/116(97[91,99]) | 22/71(31[21,43]) | 84/89(94[87,98]) | 31/69(45[33,57]) |
| Proportion with twice daily quinine maintenance dose |  |  |  |  |  |  |  |  |
| Survey1 | 0/154( 0[ 0, 2]) | 0/72( 0[ 0, 5]) | 0/161( 0[ 0, 2]) | 3/192( 2[ 0, 4]) | 3/94( 3[ 1, 9]) | 34/94(36[27,47]) | 1/234( 0[ 0, 2]) | 0/88( 0[ 0, 4]) |
| Survey2 | 89/146(61[53,69]) | 58/77(75[64,84]) | 116/120(97[92,99]) | 57/120(47[38,57]) | 41/67(61[49,73]) | 45/78(58[46,69]) | 36/166(22[16,29]) | 22/52(42[29,57]) |
| Survey3 | 94/132(71[63,79]) | 159/173(92[87,96]) | 53/55(96[87,100]) | 63/144(44[36,52]) | 14/46(30[18,46]) | 38/87(44[33,55]) | 19/175(11[ 7,16]) | 7/66(11[ 4,21]) |
| Survey4 | 117/152(77[69,83]) | 149/154(97[93,99]) | 72/77(94[85,98]) | 175/208(84[78,89]) | 43/76(57[45,68]) | 31/64(48[36,61]) | 35/81(43[32,55]) | 20/58(34[22,48]) |
| Proportion with quinine daily dose >=40mg/kg |  |  |  |  |  |  |  |  |
| Survey1 | 25/161(16[10,22]) | 4/79( 5[ 1,12]) | 4/168( 2[ 1, 6]) | 13/205( 6[ 3,11]) | 11/102(11[ 6,18]) | 14/101(14[ 8,22]) | 11/236( 5[ 2, 8]) | 34/125(27[20,36]) |
| Survey2 | 10/162( 6[ 3,11]) | 1/85( 1[ 0, 6]) | 1/129( 1[ 0, 4]) | 6/127( 5[ 2,10]) | 5/76( 7[ 2,15]) | 7/83( 8[ 3,17]) | 13/192( 7[ 4,11]) | 11/59(19[10,31]) |
| Survey3 | 4/144( 3[ 1, 7]) | 1/180( 1[ 0, 3]) | 0/59( 0[ 0, 6]) | 12/156( 8[ 4,13]) | 12/88(14[ 7,23]) | 14/93(15[ 8,24]) | 2/191( 1[ 0, 4]) | 6/77( 8[ 3,16]) |
| Survey4 | 0/159( 0[ 0, 2]) | 1/163( 1[ 0, 3]) | 1/85( 1[ 0, 6]) | 5/218( 2[ 1, 5]) | 6/84( 7[ 3,15]) | 4/71( 6[ 2,14]) | 1/89( 1[ 0, 6]) | 11/69(16[ 8,27]) |
| Proportion of pneumonia with a severity classification |  |  |  |  |  |  |  |  |
| Survey1 | 15/97(15[ 9,24]) | 19/189(10[ 6,15]) | 8/100( 8[ 4,15]) | 5/137( 4[ 1, 8]) | 5/18(28[10,53]) | 7/140( 5[ 2,10]) | 15/78(19[11,30]) | 10/146( 7[ 3,12]) |
| Survey2 | 98/112(88[80,93]) | 179/199(90[85,94]) | 105/105(100[97,100]) | 88/100(88[80,94]) | 21/30(70[51,85]) | 91/137(66[58,74]) | 63/77(82[71,90]) | 125/134(93[88,97]) |
| Survey3 | 109/117(93[87,97]) | 208/220(95[91,97]) | 144/153(94[89,97]) | 138/150(92[86,96]) | 4/25(16[ 5,36]) | 63/111(57[47,66]) | 80/123(65[56,73]) | 92/112(82[74,89]) |
| Survey4 | 76/81(94[86,98]) | 204/211(97[93,99]) | 106/111(95[90,99]) | 151/160(94[90,97]) | 14/85(16[ 9,26]) | 77/111(69[60,78]) | 70/145(48[40,57]) | 170/181(94[89,97]) |
| Proportion with once daily gentamicin dose |  |  |  |  |  |  |  |  |
| Survey1 | 1/99( 1[ 0, 5]) | 2/191( 1[ 0, 4]) | 2/125( 2[ 0, 6]) | 5/133( 4[ 1, 9]) | 0/21( 0[ 0,16]) | 20/183(11[ 7,16]) | 1/51( 2[ 0,10]) | 3/236( 1[ 0, 4]) |
| Survey2 | 44/57(77[64,87]) | 117/175(67[59,74]) | 47/47(100[92,100]) | 47/97(48[38,59]) | 21/29(72[53,87]) | 42/118(36[27,45]) | 47/56(84[72,92]) | 108/149(72[65,79]) |
| Survey3 | 61/78(78[67,87]) | 148/155(95[91,98]) | 35/35(100[90,100]) | 82/99(83[74,90]) | 36/56(64[50,77]) | 52/126(41[33,50]) | 41/51(80[67,90]) | 100/143(70[62,77]) |
| Survey4 | 62/75(83[72,90]) | 134/138(97[93,99]) | 41/46(89[76,96]) | 103/117(88[81,93]) | 68/90(76[65,84]) | 78/114(68[59,77]) | 72/84(86[76,92]) | 129/190(68[61,74]) |
| Proportion with gentamicin daily dose <4mg/kg |  |  |  |  |  |  |  |  |
| Survey1 | 46/99(46[36,57]) | 41/191(21[16,28]) | 21/125(17[11,25]) | 20/133(15[ 9,22]) | 13/21(62[38,82]) | 19/183(10[ 6,16]) | 7/51(14[ 6,26]) | 18/236( 8[ 5,12]) |
| Survey2 | 7/57(12[ 5,24]) | 5/175( 3[ 1, 7]) | 2/47( 4[ 1,15]) | 5/97( 5[ 2,12]) | 2/29( 7[ 1,23]) | 31/118(26[19,35]) | 12/56(21[12,34]) | 42/149(28[21,36]) |
| Survey3 | 7/78( 9[ 4,18]) | 3/155( 2[ 0, 6]) | 1/35( 3[ 0,15]) | 2/99( 2[ 0, 7]) | 4/56( 7[ 2,17]) | 32/126(25[18,34]) | 1/51( 2[ 0,10]) | 17/143(12[ 7,18]) |
| Survey4 | 1/75( 1[ 0, 7]) | 3/138( 2[ 0, 6]) | 0/46( 0[ 0, 8]) | 6/117( 5[ 2,11]) | 15/90(17[10,26]) | 8/114( 7[ 3,13]) | 5/84( 6[ 2,13]) | 12/190( 6[ 3,11]) |
| Proportion with gentamicin daily dose >=10mg/kg |  |  |  |  |  |  |  |  |
| Survey1 | 3/99( 3[ 1, 9]) | 1/191( 1[ 0, 3]) | 6/125( 5[ 2,10]) | 9/133( 7[ 3,12]) | 1/21( 5[ 0,24]) | 13/183( 7[ 4,12]) | 4/51( 8[ 2,19]) | 21/236( 9[ 6,13]) |
| Survey2 | 10/57(18[ 9,30]) | 21/175(12[ 8,18]) | 2/47( 4[ 1,15]) | 11/97(11[ 6,19]) | 2/29( 7[ 1,23]) | 15/118(13[ 7,20]) | 4/56( 7[ 2,17]) | 8/149( 5[ 2,10]) |
| Survey3 | 9/78(12[ 5,21]) | 6/155( 4[ 1, 8]) | 0/35( 0[ 0,10]) | 7/99( 7[ 3,14]) | 5/56( 9[ 3,20]) | 7/126( 6[ 2,11]) | 6/51(12[ 4,24]) | 21/143(15[ 9,22]) |
| Survey4 | 7/75( 9[ 4,18]) | 2/138( 1[ 0, 5]) | 3/46( 7[ 1,18]) | 9/117( 8[ 4,14]) | 13/90(14[ 8,23]) | 7/114( 6[ 3,12]) | 2/84( 2[ 0, 8]) | 31/190(16[11,22]) |
| Adequate Oxygen Prescriptions |  |  |  |  |  |  |  |  |
| Survey1 | 0/8( 0[ 0,37]) | 0/36( 0[ 0,10]) | 0/17( 0[ 0,20]) | 0/30( 0[ 0,12]) | 0/1( 0[ 0,98]) | 0/17( 0[ 0,20]) | 0/8( 0[ 0,37]) | 0/52( 0[ 0, 7]) |
| Survey2 | 2/22( 9[ 1,29]) | 15/46(33[20,48]) | 2/17(12[ 1,36]) | 0/42( 0[ 0, 8]) | 2/5(40[ 5,85]) | 0/46( 0[ 0, 8]) | 10/20(50[27,73]) | 1/77( 1[ 0, 7]) |
| Survey3 | 0/34( 0[ 0,10]) | 33/51(65[50,78]) | 2/19(11[ 1,33]) | 5/62( 8[ 3,18]) | 1/12( 8[ 0,38]) | 0/36( 0[ 0,10]) | 4/19(21[ 6,46]) | 0/72( 0[ 0, 5]) |
| Survey4 | 3/33( 9[ 2,24]) | 38/49(78[63,88]) | 6/25(24[ 9,45]) | 19/51(37[24,52]) | 0/15( 0[ 0,22]) | 1/18( 6[ 0,27]) | 1/27( 4[ 0,19]) | 0/60( 0[ 0, 6]) |
| Proportion of diarrhoea/ dehydration diagnosis with a severity classification |  |  |  |  |  |  |  |  |
| Survey1 | 27/57(47[34,61]) | 25/42(60[43,74]) | 21/57(37[24,51]) | 27/41(66[49,80]) | 7/10(70[35,93]) | 17/35(49[31,66]) | 33/48(69[54,81]) | 40/73(55[43,66]) |
| Survey2 | 87/90(97[91,99]) | 45/47(96[85,99]) | 85/85(100[96,100]) | 45/47(96[85,99]) | 19/21(90[70,99]) | 50/59(85[73,93]) | 49/54(91[80,97]) | 84/88(95[89,99]) |
| Survey3 | 63/66(95[87,99]) | 79/82(96[90,99]) | 60/60(100[94,100]) | 67/67(100[95,100]) | 10/17(59[33,82]) | 45/63(71[59,82]) | 32/40(80[64,91]) | 73/79(92[84,97]) |
| Survey4 | 57/58(98[91,100]) | 115/119(97[92,99]) | 109/110(99[95,100]) | 101/102(99[95,100]) | 28/41(68[52,82]) | 58/62(94[84,98]) | 58/68(85[75,93]) | 130/141(92[86,96]) |
| Correct fluid prescription |  |  |  |  |  |  |  |  |
| Survey1 | 2/33( 6[ 1,20]) | 2/16(13[ 2,38]) | 0/85( 0[ 0, 4]) | 3/28(11[ 2,28]) | 0/9( 0[ 0,34]) | 3/25(12[ 3,31]) | 11/27(41[22,61]) | 1/14( 7[ 0,34]) |
| Survey2 | 43/77(56[44,67]) | 9/27(33[17,54]) | 45/58(78[65,87]) | 7/29(24[10,44]) | 5/14(36[13,65]) | 0/15( 0[ 0,22]) | 12/43(28[15,44]) | 7/61(11[ 5,22]) |
| Survey3 | 29/47(62[46,75]) | 20/49(41[27,56]) | 11/24(46[26,67]) | 15/37(41[25,58]) | 3/15(20[ 4,48]) | 7/40(17[ 7,33]) | 16/40(40[25,57]) | 23/60(38[26,52]) |
| Survey4 | 28/53(53[39,67]) | 57/69(83[72,91]) | 50/65(77[65,86]) | 39/69(57[44,68]) | 8/25(32[15,54]) | 16/47(34[21,49]) | 45/66(68[56,79]) | 27/96(28[19,38]) |
